# Health care prioritization: a clinician’s duty

**DOI:** 10.1186/s40697-014-0027-4

**Published:** 2014-10-28

**Authors:** Lianne Barnieh, Cam Donaldson, Braden Manns

**Affiliations:** Department of Medicine, University of Calgary, Calgary, AB T2N 2T9 Canada; Yunus Centre for Social Business & Health, Glasgow Caledonian University, Glasgow, Scotland; Institutes for Applied Health Research and Society & Social Justice Research, Glasgow, Scotland; Department of Community Health Sciences, University of Calgary, Calgary, Canada; Institute for Public Health, University of Calgary, Calgary, Canada

**Keywords:** Health care prioritization, Clinician, Health economics, Nephrology

## Abstract

**Purpose of review:**

Publicly funded health care systems are increasingly confronted with fiscal and demographic challenges and face pressure to constrain resource use without impacting clinical outcomes.

**Findings:**

Clinicians routinely make decisions in the care of their patients that use finite health care resources. Aligning the goal of caring for their patients with ensuring that effective interventions are available for patients who are most likely to benefit is critical to sustaining the publicly funded health care system.

**Implications:**

Balancing the needs of patients with health care prioritization will require changes to be made across the health care system. Incorporating costs and value for money when caring for patients and making decisions will play an important role in efficiency and value in the health system.

## ABRÉGÉ

**Objectif:**

Les systèmes publics de santé sont de plus en plus confrontés aux défis fiscaux et démographiques, et se voient contraints de limiter l’utilisation des ressources sans toutefois compromettre les résultats cliniques.

**Résultats:**

Les cliniciens prennent régulièrement des décisions relatives aux soins aux patients qui requièrent des ressources limitées en soins de santé. Poursuivre l’objectif de prodiguer les soins aux patients, tout en assurant une offre d’interventions efficaces à ceux qui sont les plus susceptibles d’en bénéficier, est essentiel au maintien du système public de santé.

**Implications:**

Trouver l’équilibre entre les besoins des patients et l’ordre des priorités des soins de santé requerra que soient apportés des changements dans le système de santé. L’intégration des coûts et de l’optimisation des ressources lors de la prestation des soins aux patients et de la prise de décisions jouera un rôle important dans l’efficacité et la valeur du système de santé.

## Why is this report important

Publicly funded health care systems face increasing pressure to constrain resource use without impacting health care services. Clinicians are a key member of the health care team, being responsible for ordering tests and treatments.

## What are the key messages

Most decisions regarding clinical care should be made after consideration of potential benefits and costs, incorporating the notion of value for money. Health care prioritization should be a duty for all involved in the care of patients, and active clinician engagement is required.

## Introduction

Confronted with fiscal and demographic challenges [1], publicly funded health care systems face increasing pressure to constrain resource use without impacting health care services. As less than one half of one percent of the nation’s population [2], clinicians, be it a nephrologist or an allied health professional caring for someone with kidney disease, determine how over 10% of the gross domestic product is spent [3], making clinical decisions “purchasing decisions”. These decisions should be made within the context of competing uses of finite resources to ensure that the most effective interventions are available for the patients who are most likely to benefit [4]. Health care managers indirectly determine how to allocate scarce health care resources, by determining what tests and treatments will be made available. However, prioritizing health care resources should not be seen as the sole responsibility of managers and decision makers, and to be successful in maximizing population health, active engagement of clinicians, who directly allocate health care resources through ordering of tests and treatments, is required.

The issue of health care prioritization is particularly relevant for clinicians working with patients with kidney failure since they are often cared for in the context of provincial renal programs, who determine how allocated resources are best used to care for all patients with end-stage renal disease (ESRD). In this context, funds or resources spent on one patient are no longer be available for other patients with kidney failure in the renal program. Herein, we discuss the challenges that clinicians face in participating in health care prioritization and outline how they can incorporate the cost of health care and the concept of value for money in their decision making.

## Review

Clinicians (the providers) receive extensive training in how to investigate, diagnose, and manage patients, but receive little training in health care priority setting. On the other hand, health care systems and administrators (the payers) focus almost exclusively on priority setting, outlining overall objectives for the system. Their mandate stresses staying within allocated budgets, and though they have a lot of responsibility, they typically lack the power to affect the decisions made on the front-lines by clinicians. While most private enterprises are able to direct the performance of their employees through incentives to ensure their goals are met, the Canadian health care system is not a private enterprise and most nephrologists in Canada are independent practitioners. Clinicians’ are broadly motivated by one of three things: the benefit to the patient (i.e. successful transplant), social good (i.e. promoting a healthy lifestyle), and/or personal interests and desires (i.e. target income) [5]. Clinicians are accountable to their patients by ensuring that the care they provide serves the best interest of their patients. However, they are also accountable to the greater health care system and by extension to other patients, for their spending patterns. Clinicians have two fundamental responsibilities: to “consider the well-being of the patient” and to “consider the well-being of society in matters affecting health” [6]. Dealing with a potential lack of alignment between the priorities of clinicians and the health system is critical to addressing the common objectives of both parties: providing high quality health care and improving outcomes for all, maximizing beneficence and justice.

### Suggestions for incorporating cost and value for money within health care

To ensure health system sustainability, changes are needed, both for clinicians and for the broader health system (Table 1).

**Table 1:** **Proposed strategies to consider costs and the concept of value for money when caring for patients**

**Proposed strategy**	**Example**
Continuing medical education should educate clinicians on how to incorporate cost and “value for money” in clinical practice, particularly for management of common clinical conditions	Educating clinicians to promote home dialysis options, given the cost savings and equal or better outcomes for patients that are eligible
Emphasize that considering treatment costs is particularly important in the face of clinical uncertainty	Routine management of abnormalities of mineral metabolism with expensive medications with an incomplete evidence base such as non-calcium based phosphate binders and cinacalcet
Consider the impact on health care costs when developing clinical practice guidelines	During the development of the timing of dialysis initiation guidelines by the Canadian Society of Nephrology [7], resource use was incorporated as a secondary outcome
Ensure that clinician leaders are engaged when developing local health policies that incorporate costs within renal programs	Developing clinical practice guidelines to increase the use of home therapies
	Support knowledge dissemination activities related to existing clinical practice guidelines

### Suggestions for clinicians

Firstly, the way future clinicians are educated needs to be re-evaluated. Medical students and residents need to be taught how to reasonably incorporate the notion of “value for money” into their daily care for patients. An understanding of basic principles of health economics, and health care prioritization is required—fundamental skills that should be part of undergraduate and continuing medical education. While being a health care manager is a core competency within CanMEDS, even the basics of how to incorporate the notion of cost into clinical decision-making is not emphasized during formal education of undergraduate students or residents and is not emphasized during continuing medical education [8]. Medical students and residents should be taught not only how to integrate cost into their practice, but the principles of economic evaluations, including how to read and interpret economics evaluations for common treatments in their practice area [9]. Clinicians make value-based decisions about other finite resources such as their time and use of beds in a hospital or intensive care unit—and need to apply this same skill set to other health care resources [10]. A clinician with the required skill set to participate in priority setting in an informed way, is more likely to participate, benefiting the patient and the system.

Although rarely discussed during training, it should be made clearer that in a cash strapped system, resources may be redeployed to fund more effective interventions. This is particularly true if the effectiveness of the intervention is marginal at best, or has only been shown to impact nonclinical endpoints [11], in other words, when significant clinical uncertainty exists. Clinicians should not feel compelled to routinely offer such therapies or tests, particularly when they are notably more expensive than the current standard of care. Clinicians need to begin to routinely consider cost and “value for money” in clinical practice, particularly when making management decisions for situations where several potential strategies exist.

Value for money is usually estimated using economic evaluations, which weigh the resources used (costs) with health outcomes (benefits) of competing programs. This is central to health economics and rests on the principles of scarcity and choice [4]. Given resource scarcity, choices must be made about what health programs or services to provide and which to forgo -- also known as “opportunity cost”. For example, when developing the budget for a renal program, money may be directed to more aggressive monitoring of vascular accesses, or to hire additional personnel to expedite the workup of potential kidney donors. If the program chose to hire additional personnel to expedite a donor’s transplant workup, the opportunity cost of this would be the clinical benefits that patients might have received had more aggressive monitoring of vascular access been implemented. When making a decision in health economics, one must ensure that the value of a treatment is greater than the “opportunity cost” (i.e. the health benefits) of other programs that are being considered. Since clinicians working in publicly funded health care systems spend limited tax-payer dollars, they should consider the resource implications of each treatment, in addition to the impact on health outcomes, by applying the above principles.

While physicians might feel uncomfortable considering health care costs in all situations, for instance when a patient’s life is in immediate risk, in most situations, considering the opportunity cost of the treatment under consideration is feasible and important. Indeed, if costs were integrated when developing clinical practice guidelines, incorporating the consideration of cost into usual clinician practice would become easier. While cost constraint is not the explicit goal of the Choosing Wisely campaign, this is an example of an initiative (currently being undertaken within nephrology in Canada by the Canadian Society of Nephrology) which is meant to help clinicians and patients engage in conversations about unnecessary tests, treatments and procedures in order to make smart and effective choices in care [12].

Lanthanum carbonate is one example of the issue faced by clinicians when costs are not incorporated when developing clinical practice guidelines. Lanthanum carbonate was reviewed for consideration of funding by the Canadian Common Drug Review (CDR) [13] in 2008. Eight RCTs in a total of 2,646 patients, ranging in duration from four weeks to two years, met the inclusion criteria for the systematic review. In these studies, lanthanum was not shown to improve quality of life, or reduce rates of bone fracture or cardiovascular complications. Acknowledging that lanthanum has been shown to cause fewer episodes of hypercalcemia than calcium-based phosphate binders, given a cost per day of lanthanum between $6.18 to $12.23, the CDR did not recommend funding for lanthanum. However, this decision is not consistent with contemporary clinical practice guidelines for the management of bone and mineral metabolism abnormalities in kidney disease which recommend the use of non-calcium containing phosphate binders in several clinical scenarios, including as first-line therapy [14]. Without incorporating the impact of treatment on costs in the guidelines, it is difficult for clinicians to incorporate the notion of “value for money” into their routine practice, and inevitably leads to conflict between prescribers and health system funders.

As leaders within the health care system, though clinicians may be reluctant to incorporate the consideration of cost in their daily care, their role in allocating scarce resources cannot be avoided. Indeed, since one of the roles of publicly funded health care is to maximize health gains within a restricted budget, limiting expensive therapies to those who can benefit most seems a reasonable and equitable approach. Clinician leaders can be engaged to act on behalf of other clinicians when establishing health policy, including determining what new programs to offer, particularly in nephrology, given that care of patients with kidney failure is often funded within provincial renal programs. Within such programs, modifying and adhering to clinician-developed clinical practice guidelines that take cost into account could help ease the tension between clinician’s clinical decision making and health system objectives.

### Suggestions for the broader health system

Table 2 highlights ways in which cost can be considered within the health care system.

**Table 2:** **Proposed changes to the broader health care system to increase consideration of costs and the concept of value for money when caring for patients**

**Proposed change**	**Who can make the change**
Incorporate health economics and consideration of cost into medical education as a professional ethic	Royal College of Physicians and Surgeons of Canada
	Medical schools
Similar to the system that currently exists for assessing new medications [13], when assessing new tests or technologies for funding, health systems should systematically review evidence on the impact on outcomes and costs, providing preferential access to those tests and technologies offering best value for money (i.e. better outcomes for less money)	Provincial health systems
	Federal health ministry
To align physician activities with health system goals, consider the optimal mix of payment methods for physicians	Provincial health systems
	Provincial medical associates

Clinicians are not the only group that needs to be targeted to offload stress in the system; change by other health care players could make it easier for physicians to consider value for money. For instance, to achieve a sustainable health care system, decision makers need to systematically consider information on effectiveness and costs when determining what new tests and treatments to offer. While this process is entrenched within the assessment of new drugs across Canada [13], it is not yet systematically applied to other non-drug interventions before they are made available across Canada. To assist with larger and more complex health programs, the process of program budgeting and marginal analysis (PBMA) is used for priority setting, and may help align the goals of doctors and managers by continually assessing and re-assessing marginal effectiveness of available programs [15]. PBMA examines programs – for either the same or different groups of patients - that are either close to being funded, or of limited value where the program is considered for retraction, assessing whether the benefits of the new program added outweigh the benefits an existing program that costs a similar amount.

Another issue worth considering is that most physicians in Canada are still remunerated on a fee-for-service basis, which is appropriate in areas where the health care system has high volume needs, such as vaccination and preventive care, but may be less appropriate for some areas within nephrology (for instance, payment for caring for patients with ESRD on dialysis). Though physician remuneration is an important issue, governments appear unwilling to deal with this highly contentious issue. Alternative mechanisms to pay physicians, without altering their employment status as an independent practitioner, exist. In England, only about 10 to 15% of physician salaries are fee-for-service, with the remainder based on capitation and targeted payments [4]. Designing a remuneration system which is consistent with the objectives of the health care system, but provides physicians independence all while offering acceptable incentives to reward priority activities is, therefore, challenging, but feasible.

## Conclusions

Balancing the needs of patients with health care prioritization may be one of the most complex and challenging tasks facing today’s clinician, but one that needs to be urgently undertaken if publicly funded health care is to be sustained. As clinicians play an important role in efficiency and value in the health system, creating successful partnerships between clinicians and health care managers is crucial to aligning the goals of both parties and cultures.
